# Editorial: Optogenetic and chemogenetic insights on sensory stimulus detection to motivated behaviors and reinforcement

**DOI:** 10.3389/fncir.2023.1177498

**Published:** 2023-04-25

**Authors:** Anton Ilango, T. Chase Francis, Mary Kay Lobo

**Affiliations:** ^1^Helmholtz Institute for Metabolic, Obesity and Vascular Research, Helmholtz Center München, Helmholtz Association of German Research Centres (HZ), Leipzig, Germany; ^2^Department of Drug Discovery and Biomedical Sciences, College of Pharmacy, University of South Carolina, Columbia, SC, United States; ^3^Department of Anatomy and Neurobiology, University of Maryland School of Medicine, Baltimore, MD, United States

**Keywords:** optogenetics, chemogenetics, reward, feeding, motivation

The arrival of optogenetics and chemogenetics have given neuroscientists an unprecedented ability to study the neural circuits from the genetic to cellular level. While differing in timing, targeted manipulation, controlling stimulation and invasiveness, both methodologies have helped neuroscientists target and control neural activity. These methodologies have allowed neuroscientists to construct a detailed view of circuits underlying motivation, reward, learning, neuropsychiatric, and neurodegenerative disorders. The present Research Topic is built upon our previous one “*Neural circuits underlying emotion and motivation: Insights from optogenetics and pharmacogenetics*” in Frontiers in Behavioral Neuroscience and provide additional insights into the recent progress.

In this Research Topic, two primary research articles explored primary sensory cortical neurons of the auditory and somatosensory cortices. The article by Cheng et al. studied how paired stimulation promotes neuroplasticity in primary somatosensory cortex. They paired the optogenetic stimulation of primary somatosensory cortex and whisker deflection with *in vivo* neuronal activity recordings to find the optimum parameters to manipulate feature preferences in barrel cortical neurons. They reported a temporally selective protocol for pairing optogenetic-mechanical stimulation that induced *in vivo* neuroplasticity and feature selectivity.

In another article, Weible et al. addressed the role of Gpr26 neurons in sound detection, particularly in detection of gaps in sound to better understand their role in temporal processing of the auditory system. Predominantly expressed in layer 4, Gpr26 neurons are known for detecting the gaps in background noise and facilitate the pre-pulse inhibition of the acoustic startle response. Weible et al., concluded that photo-stimulation was sufficient in attenuating the startle reflex and suppression led to reduction of *in vivo* auditory cortical firing to gaps and impaired behavioral gap detection.

During energy deficit, agouti-related peptide (AGRP)-expressing neurons in the arcuate nucleus (ARC-AGRP neurons) express higher level of activity, which regulate the feeding behavior. ARC-AGRP ablation leads to severe body weight reduction and food intake. Opioids are known to act on brain regions that governs reward and feeding and interact with AGRP systems in complex ways. Here, Laing et al. addressed whether the effects of morphine administration affects feeding and body weight changes induced by chemogenetic activation of ARC-AGRP neurons. They demonstrated how ARC-AGRP neurons can influence opioid effects on weight maintenance.

Circuits underlying reward and aversion are interconnected and their connections have been teased apart by combinatorial methods using optogenetics. Here, the review by Chen highlights the recent progress made on the role of network of brain regions such as ventral tegmental area (VTA), nucleus accumbens and basal forebrain areas in reward and aversion. It describes circuit mechanisms that contribute to processing of reward and aversive signals.

A review by Soares-Cunha and Heinsbroek explicitly details the function of the ventral pallidum (VP) which is considered as a central node of the ventral part of the basal ganglia. This review extensively covers the role of the VP in regulating motivation, reward, and aversion, with a special emphasis on the cellular heterogeneity and circuit-specific regulation of motivated behaviors underlying mood and substance use disorders.

We believe that these powerful methods will continue to have a strong influence on our ability to decipher brain function and connectivity. We hope you will enjoy reading our updated Research Topic “*Optogenetic and chemogenetic insights on sensory stimulus detection to motivated behaviors and reinforcement*.”

## Author contributions

AI initiated the Research Topic. MKL and TCF co-hosting the Research Topic. All authors contributed to the article and approved the submitted version.

